# Cytotoxic lanthanum oxide nanoparticles sensitize glioblastoma cells to radiation therapy and temozolomide: an in vitro rationale for translational studies

**DOI:** 10.1038/s41598-020-75372-3

**Published:** 2020-10-23

**Authors:** Victor M. Lu, Toni Rose Jue, Kerrie L. McDonald

**Affiliations:** 1 Lowy Cancer Center, University of New South Wales, Sydney, NSW Australia; 2 Department of Neurological Surgery, University of Miami Miller School of Medicine, 1600 NW 10th Ave #1140, Miami, FL 33136 USA

**Keywords:** CNS cancer, Nanoparticles, Cell death

## Abstract

Glioblastoma (GBM) is a malignant brain tumour with a dismal prognosis, despite best treatment by surgical resection, radiation therapy (RT) and chemotherapy with temozolomide (TMZ). Nanoparticle (NP) therapy is an emerging consideration due to the ability of NPs to be formulated and cross the blood brain barrier. Lanthanum oxide (La_2_O_3_) NPs are therapeutically advantageous due to the unique chemical properties of lanthanum making it cytotoxic to cancers, and able to enhance existing anti-cancer treatments. However, La_2_O_3_ NPs have yet to be thoroughly investigated in brain tumors. We show that these NPs can reach the brain after venous injection, penetrate into GBM cells via endocytosis, dissociate to be cytotoxic, and enhance the therapeutic effects of RT and TMZ. The mechanisms of cell death by La_2_O_3_ NPs were found to be multifaceted. Increasing NP concentration was correlated to increased intrinsic and extrinsic apoptosis pathway markers in a radical oxygen species (ROS)-dependent manner, as well as involving direct DNA damage and autophagic pathways within GBM patient-derived cell lines. NP interactions to sensitize GBM to RT and TMZ were shown to involve these pathways by enhancing ROS and apoptotic mechanisms. We therefore demonstrate the therapeutic potential of La_2_O_3_ NPs to treat GBM cells in vitro, and encourage translational exploration in the future.

## Introduction

Of all known adult brain tumors, glioblastoma (GBM) is the most lethal. In its defining trial in 2005, chemotherapy agent temozolomide (TMZ) added to radiation therapy (RT) improved median survival to 15 months^1^. Since then, survival gains have been modest at best, and interest in novel therapies continues to grow traction in order to improve prognosis^2,3^.

Although the use of nanoparticle (NP) therapy to treat GBM remains relatively novel, there has been long-held interest in utilizing NP therapy to target cancers and improve current treatment options^4^. Specifically, they serve as nanoscale delivery vectors that can act as sensitizing agents due to intrinsic optical and electrical properties^5^. Furthermore, functionalization of these NPs can further enhance synergist properties while sparing normal healthy tissue^6^. A specific attraction for using NP therapy to target brain tumors such as GBM is the fact they have been proven to infiltrate the blood brain barrier (BBB) to reach brain matter and target tumor cells^7,8^. Yet, identifying the optimal NP composition to target GBM remains an ongoing effort to this day.

Lanthanum (La) is a rare earth element (REE), one of seventeen *f*-electropositive metallic elements. Despite the name, these elements are not particularly rare, and readily found in the human body, at intravenous levels in the order of 1 ng/kg which remain > 1000 fold below the toxicity threshold^9,10^. Studies into these REEs have previously described cytotoxic and anti-cancer effects due to its unique electronic configuration—this includes radiosensitization via the Auger effect, and chemotherapeutic synergy via increased apoptotic activity^11,12^. Despite this, lanthanum-based compounds have not been thoroughly investigated in the setting of GBM, but has two specific advantages that render it an element of interest to treat GBM. Firstly, it has been proposed that GBM cells have a natural tendency to accumulate lanthanum and its free ion La^3+^ compared to normal astrocyte cells^13^. Secondly, lanthanum forms stable oxide La_2_O_3_ that can be formulated into nanoparticles (NPs), which can be used to circumnavigate the BBB which has proven a serious barrier to otherwise promising therapeutic compounds^7,8^.

To date, lanthanum-based NP therapies have not been widely investigated in vitro for their potential to target specifically GBM and enhance RT and TMZ^12^. Correspondingly, the aim of this study was to elucidate the preliminary molecular rationale and mechanism of utilizing La_2_O_3_ NPs as a therapeutic adjunct in the treatment of GBM cells.

## Results

### La_2_O_3_ NPs enter the brain and GBM cells via clathrin-mediated endocytosis

**Figure 1:**
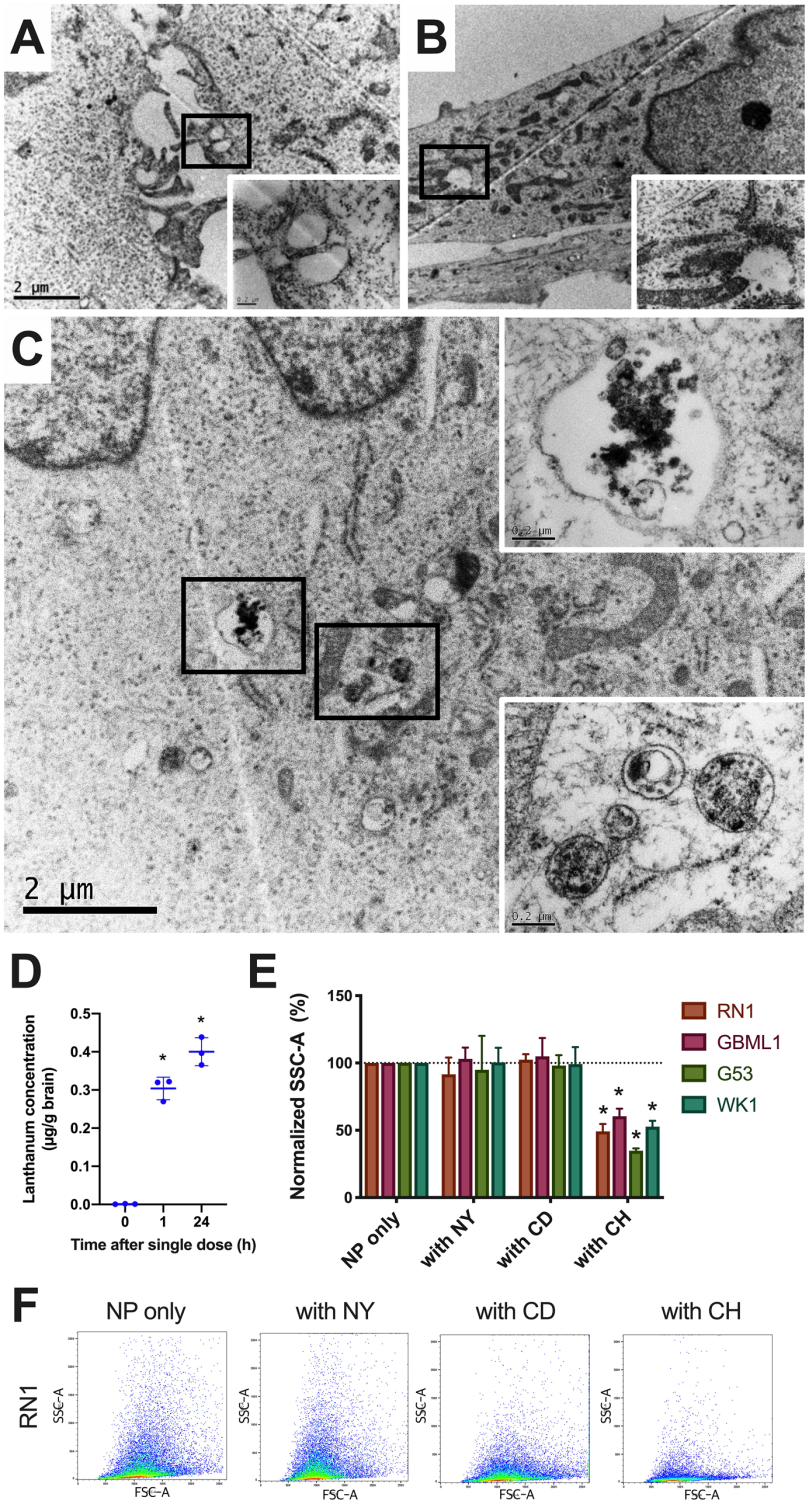
La_2_O_3_ NPs enter brain and GBM cells via clathrin-mediated pathway. Representative transmission electron microscopy images of PDCL RN1 where images (**A**) and (**B**) were not exposed to La_2_O_3_ NP, and vacant endosomes were identified. Image (**C**) was exposed to La_2_O_3_ NP for 1 h, and NPs were identified in both early (lower box) and late (upper box) endosomes in the cell. (**D**) Significant amounts of lanthanum were found in the brain 1 h and 24 h by means of mass spectrometry after NP injection (5 mg/kg) into tail vein of balb/c nude mice (n = 3 per group). Using a series of inhibitors (nystatin, NY; cytochalasin D, CD; chlorpromazine, CH), endocytosis mechanisms of NPs were then investigated (**E**) across four PDCLs, with (**F**) representative results. Cytometry data were obtained in technical triplicate over three independent tests. Values presented as mean ± SD, and flow cytometry plots are presented as forward scatter area (FSC-A, x-axis) vs side scatter area (SSC-A, y-axis). Statistical analysis was performed using two-way ANOVA analysis, with significance set at *P* < 0.05 (*).

Using transmission electron microscopy, we demonstrated NP aggregation in early- and late-stage endosomes in GBM cells 1 h after exposure (Fig. 1A–C). Its translation into practice was affirmed by detection in the brain of lanthanum 1 and 24 h after NPs were administered to the tail vein of balb/c nude mice (Fig. 1D). Flow cytometry analysis demonstrated the combination of NP and chlorpromazine was the only one to lead to statistically significant reductions in side-scatter area across four patient derived cell lines (PDCLs; RN1, GBML1, G53, WK1), which is known to specifically inhibit clathrin-mediated endocytosis (Fig. 1E–F).

### La_2_O_3_ NPs generate free La^3+^ ions and are cytotoxic to GBM cells via radical oxygen species

**Figure 2:**
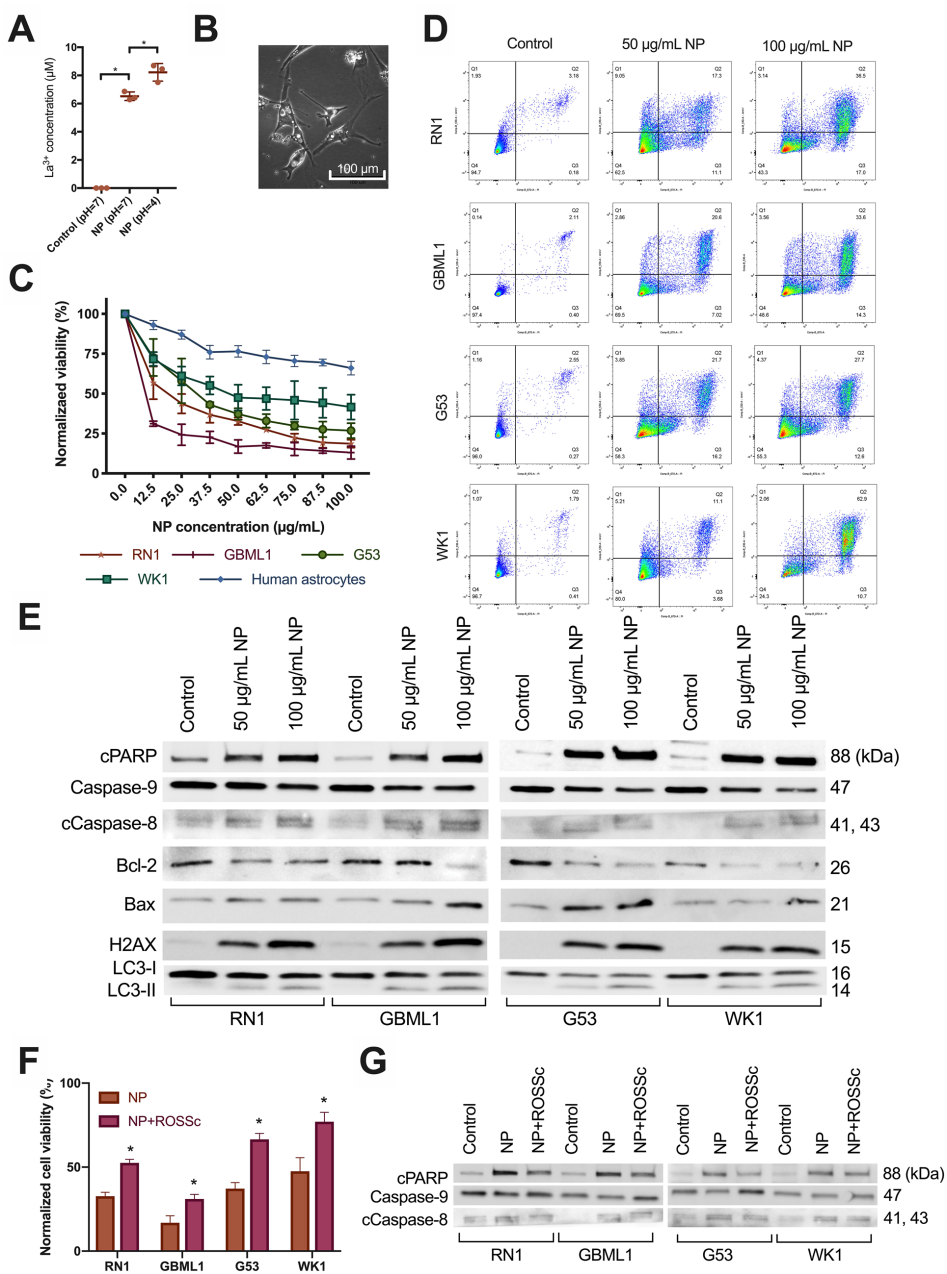
La_2_O_3_ NPs generate La^3+^ ions and cause cell death in GBM cells via a ROS mechanism. (**A**) Evidence of increased free La^3+^ ions in supernatant of 72 h NP solution (1000 µg/mL) at pH = 4 versus pH = 7 versus control (n = 3 per group). Mean differences to control were 6.5 and 8.2 µM respectively. Statistical analysis was performed using student t-test analysis, with significance set at *P* < 0.05 (*). (**B**) Light microscopic evidence of vacuolization and cell death in PDCL WK1 after 72 h of exposure to NP (100 µg/mL) at 40 × magnification. (**C**) Cytotoxicity of La_2_O_3_ NPs in PDCLs after 72 h across a concentration range of 0–100 µg/mL with relevance of (**D**) the apoptotic and autophagic pathways shown in each PDCL and (**E**) representative western blotting trends indicating intrinsic, extrinsic, and autophagic processes were involved in cell death; cleaved PARP (cPARP), Caspase-9, cleaved Caspase-8 (cCaspase-8), Bcl-2 and Bax; autophagy-related proteins LC3-I and LC3-II; DNA-damage-related ɣ-H2AX. (**F**) Significantly increased cell viability across all PDCLs 72 h after NP (50 µg/mL) was shown after 1 h pretreatment with radical oxygen species scavenger (ROSSc) with (**G**) representative western blotting trends showing reduction of cPARP, Caspase-9 and cCaspase-8 changes following pretreatment with ROSSc. Full blots provided in Supplementary. All cell viability data were obtained in technical triplicate over three independent tests and presented as mean ± SD. Statistical analysis was performed using two-way ANOVA analysis, with significance set at *P* < 0.05 (*).

Although La_2_O_3_ is relatively stable, we showed that significant amounts of free La^3+^ ions were detectable after 72 h in supernatant of NP suspensions by means mass spectrometry, and were and proportional to pH (Fig. 2A). Evidence that cell death processes such as vacuolization were occurring 72 h after NP exposure could be seen via light microscopy (Fig. 2B). All PDCLs were exposed to NPs at concentrations ranging for 0–100 µg/mL and cell viability was measured after 72 h. All demonstrated cytotoxic trends with increasing NP concentration, which were significantly lower than what was observed in normal human astrocyte cells (Fig. 2C). At the 100 µg/mL concentration, final cell viabilities were 19% in RN1, 13% in GBML1, 27% in G53, and 42% in WK1.

The primary mechanisms of cell death were suspected to be autophagy and apoptosis based increasing proportions of early and late apoptotic cells corresponding to increased NP concentration by Annexin V/PI staining (Fig. 2D). These suspicions were confirmed by increasing apoptotic cPARP and autophagic LC3-I and LC3-II expression with increasing with concentrations of NP across all PDCLs (Fig. 2E).

Evidence of intrinsic and extrinsic apoptosis pathway involvement was indicated by decreasing Caspase-9 and increasing cleaved Caspase-8 expressions respectively following increasing concentrations of NP across all PDCLs (Fig. 2E). The involvement of the intrinsic pathway was further confirmed with corresponding mitochondrial changes of increasing Bax and decreasing Bcl-2 expressions following increasing concentration of NP across all PDCLs. Finally, direct double-strand DNA damage was indicated as another involved pathway with increasing ɣ-H2AX following increasing concentration of NP across all PDCLs.

One hour pretreatment by a radical oxygen species (ROS) scavenger (ROSSc) before 72 h exposure to NP significantly increased cell viability across all PDCLs (Fig. 2F), and trends previously observed via western blotting for cPARP, Caspase-9 and cleaved Caspase-8 trended in the reverse (Fig. 2G), indicating the involvement of ROS in cytotoxicity of these NPs.

### La_2_O_3_ NPs augment radiation via cellular ROS mechanisms

**Figure 3:**
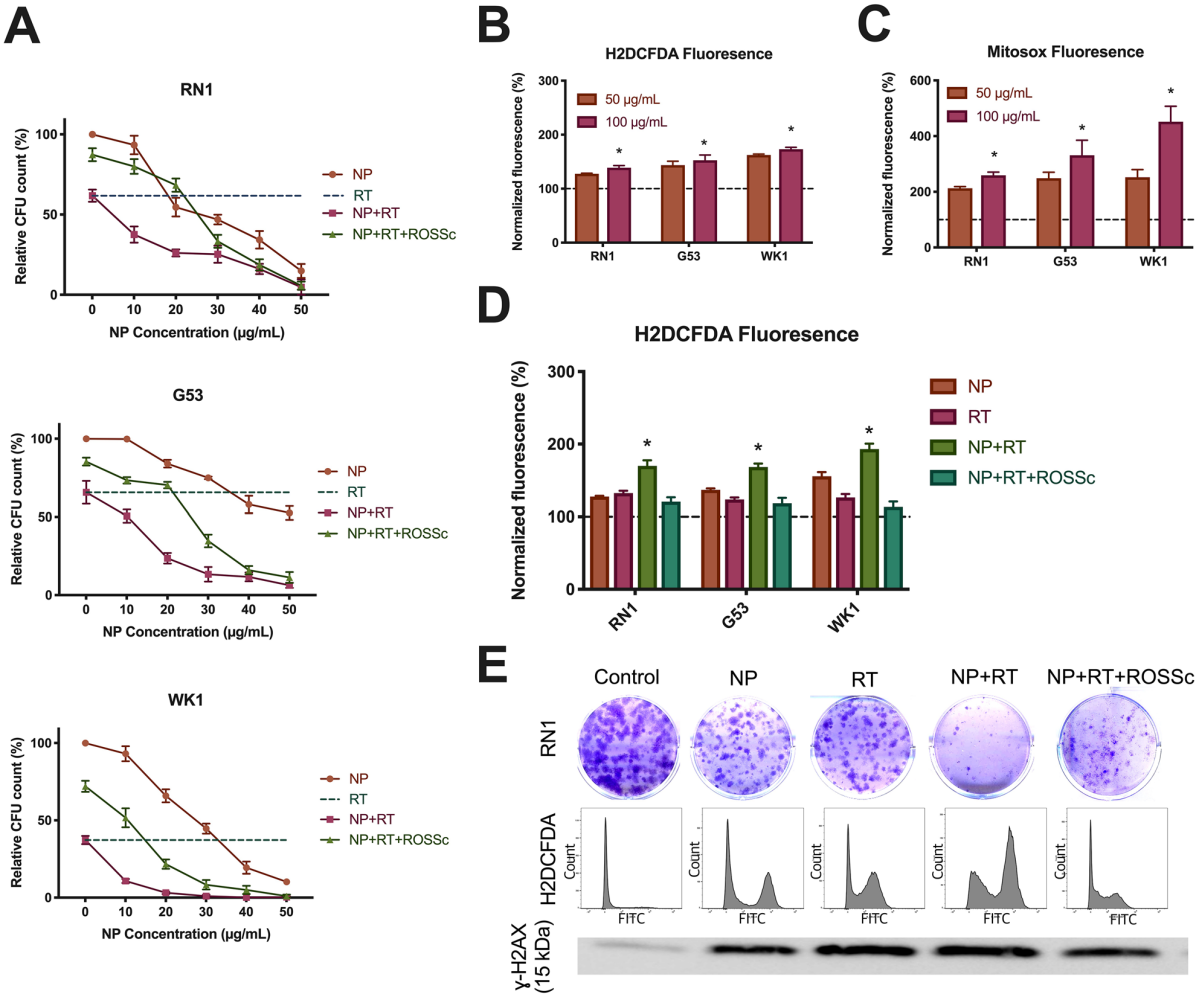
La_2_O_3_ NPs sensitize GBM to radiation via a ROS mechanism (**A**) Significantly decreased number of colony forming units (CFUs) in colony-forming PDCLs 14 days after exposure to 4 Gy radiation therapy (RT) given 1 h after NP (50 µg/mL) administration, which was partially rescued by 1 h pretreatment with ROS scavenger (ROSSc). The generation of ROS was shown in this set-up by (**B**) general intracellular fluorescent marker H2DCFDA and (**C**) mitochondria specific fluorescent marker Mitosox. (**D**) Augmentation of ROS generation by NP of RT in this set-up was then demonstrated using H2DCFDA, which was significantly reduced by pretreatment with ROSSc. (**E**) Representative western blotting of increased ɣ-H2AX expression when combining NP and RT which reduced by pretreatment with ROSSc, which correlated to clonogenic assay and flow cytometry results of H2DCFDA in PDCL RN1 (FITC, fluorescein isothiocyanate vs count). Full blots provided in Supplementary. All CFU and fluorescence data were obtained in technical triplicate over three independent tests and presented as mean ± SD. Statistical analysis was performed using two-way ANOVA analysis, with significance set at *P* < 0.05 (*).

The potential for La_2_O_3_ NP therapy to impact the effect of RT on cell viability was validated in colony-forming PDCLs RN1, G53 and WK1 at 4 Gy, after confirming decreased colony forming units (CFU) with increasing RT dose in the presence of NPs (Supp Fig. 1). We observed significant reduction in relative 14-day number of CFUs in the combination of La_2_O_3_ NP + RT compared to RT alone in all PDCLs at multiple NP concentrations, with evidence of rescue following 1 h pretreatment by ROS scavenger (ROSSc) (Fig. 3A). To determine potential triggers for RT augmentation, the generation of ROS after exposure to the La_2_O_3_ NP therapy was investigated by means of flow cytometry. Expression of intracellular ROS marker H2DCFDA was significantly increased with increasing concentrations of NP across all PDCLs (Fig. 3B). To further localize ROS generation, ROS generation within the mitochondria was then evaluated by Mitosox, which was significantly increased with increasing concentrations of NP across all PDCLs (Fig. 3C).

Given the relationship between ROS generation following RT, the potential for the La_2_O_3_ NP therapy to augment the ROS produced by RT was investigated by means of flow cytometry. There were significant increases in general ROS marker H2DCFDA in the NP + RT combination compared to NP only and RT only across all PDCLs, which were attenuated by ROSSc pretreatment (Fig. 3D). Finally, to clarify the possible downstream interaction between La_2_O_3_ NP therapy and RT, the expression of dsDNA damage marker ɣ-H2AX was also investigated by means of western blotting, showing increased expression in the NP + RT combination compared to NP only and RT only, which was attenuated by ROSSc pretreatment (Fig. 3E).

### Synergy with temozolomide is modulated by apoptosis pathway

**Figure 4:**
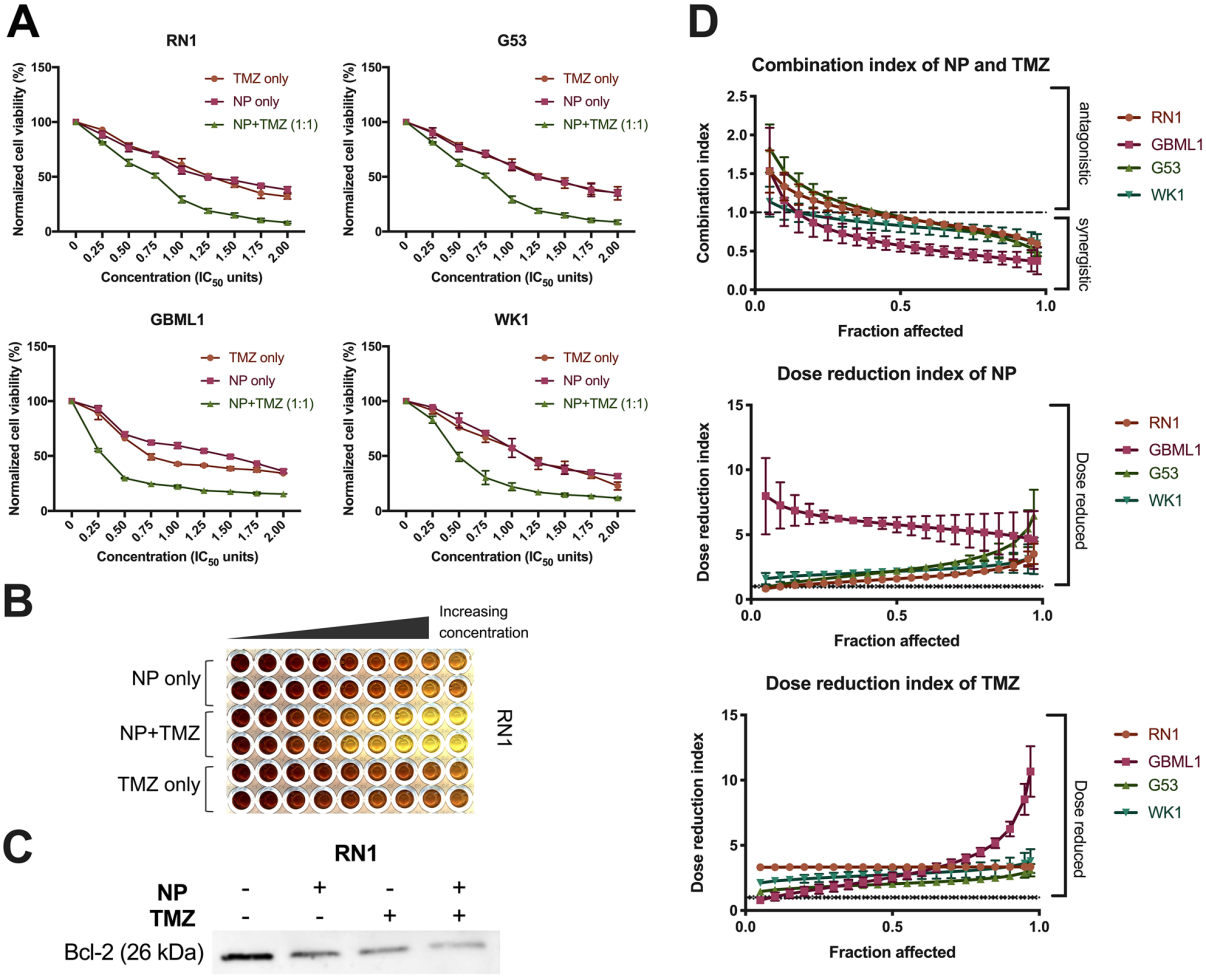
La_2_O_3_ NPs sensitize GBM to temozolomide via an apoptotic mechanism. (**A**) Increased cell death 72 h following concurrent NP and temozolomide (TMZ) at various IC50 multiples with (**B**) a representative image. (**C**) Representative western blotting of decreased bcl-2 expression with NP (50 µg/mL) and TMZ (200 µM) in PDCL RN1 was then correlated to the outcomes of the combination studies. Full blots provided in Supplementary. (**D**) Combination analysis of cell viability data per the Chou and Talalay method modelled combination indices (CIs) and dose reduction indices (DRIs) across affected fraction (F_a_) 5–97%. All cell viability data were obtained in technical triplicate over three independent tests and presented as mean ± SD. Statistical analysis was performed using two-way ANOVA analysis, with significance set at *P* < 0.05 (*).

The synergistic effects of La_2_O_3_ NP and TMZ was examined, using a range from 0.25–2.00 × IC_50_ values for an 8-day treatment period per our group’s protocol (Supp Fig. 2). La_2_O_3_ NP and TMZ were administered separately, and in fixed 1:1 ratio together, to all PDCLs (Fig. 4A,B). There was a significant decrease in relative cell viability observed in NP + TMZ combination when compared to TMZ only in all PDCLs.

These results were then modelled by combination analysis with Compusyn software (ComboSyn, Paramus, USA). Combination indices (CIs) were then modelled per the Chou and Talalay method (Fig. 4C). At 97% fraction affected (Fa), mean CIs for RN1, G53, GBML1, and WK1 were all < 1 implying synergy across all PDCLs. Furthermore, combination dose-reduction indices (DRIs) were obtained. At Fa = 97%, mean DRIs for NP and TMZ were all > 1, implying significant dose-reductions for both NP and TMZ.

To determine downstream interaction between the La_2_O_3_ NP and TMZ, anti-apoptotic protein bcl-2 expression was investigated by means of western blotting (Fig. 4D). There was decrease in bcl-2 expression in the NP + TMZ combination compared to NP only and CT only suggesting modulation of apoptotic pathways was likely involved in the observed synergy.

## Discussion

**Figure 5:**
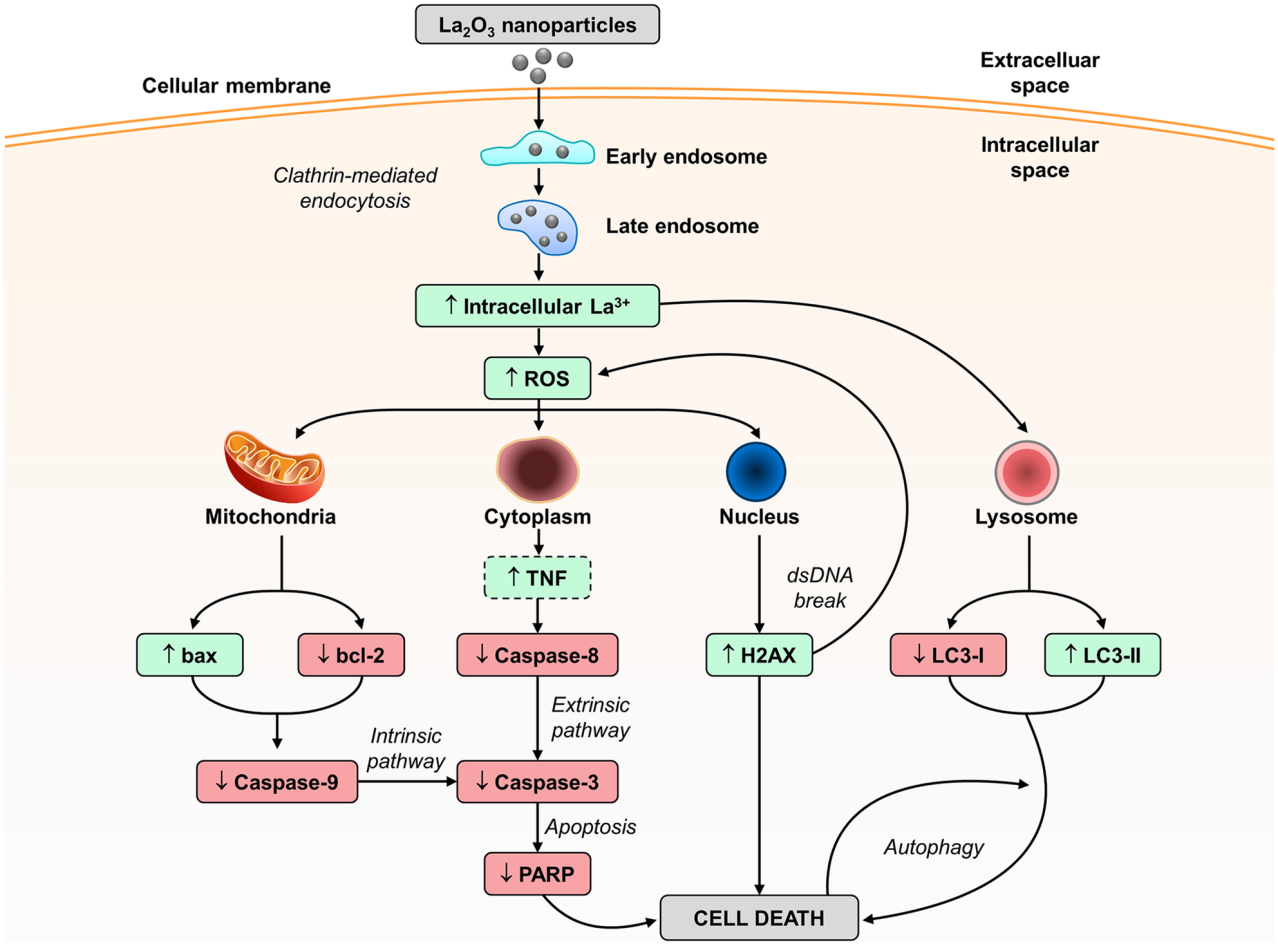
The proposed mechanisms of cell death in GBM La_2_O_3_ NP therapy. Once NPs enter the cell by endocytosis, they localize to a number of potential organelles, and induce one of three possible pathways to cell death; (1) apoptosis, intrinsic and extrinsic; (2) direct double stranded DNA (dsDNA) damage; and (3) autophagy. Changes to protein expression were categorized into increase (green) or decrease (red) after exposure to the NP therapy. Interactions with radiation therapy and chemotherapy temozolomide were shown to promote the ROS/ɣ-H2AX and bcl-2 trends respectively. Increase in tumor necrosis factor (TNF) was assumed (dotted square). Figure created using Microsoft PowerPoint (Microsoft, US). LC3; light chain 3; PARP, Poly (ADP-ribose) polymerase; ROS, reactive oxygen species.

We present the first molecular rationale and mechanism (Fig. 5) for La_2_O_3_ NP therapy as a potential option to fill this void, which takes advantage of NP biodistribution and REE biochemistry, to target GBM in the brain and augment current treatment options. A large barrier in modern day GBM therapeutic research is overcoming the BBB to target the brain and then infiltrate the cells. We not only show with preliminary efforts that these NPs can reach the brain from an intravenous injection, but also GBM cells take these NPs up. Prior to our study, only non-lanthanum REE NP therapies have been investigated in GBM, in which clathrin-mediated endocytosis was also shown to be the primary uptake pathway^14^. This echoes much of the REE NP reports in other cells, such as normal keratinocytes, as well as cancer cells of the colon and ovaries^15,16^.

Our previous efforts^17^ highlighted the potential for cytotoxicity by La_2_O_3_ NPs in immortalized GBM cell lines, which we validate in PDCLs in this current study. Furthermore, our observations correlate with broader literature where lanthanum-based NP therapies have been shown to be cytotoxic across a number of cancers other than GBM^18,19^. The reason for this cytotoxicity is likely that the La^3+^ ionic radii is the closest in size to that of Ca^2+^, an integral component of the intracellular machinery^17^. Although relatively stable, we show that La_2_O_3_ NPs can dissociate into free La^3+^ ions that can then go onto mimic Ca^2+^_,_ and interfere with standard cellular processes necessary for viability^20,21^. The consequences of these disruptions could be further enhanced in the setting of tumors, such as GBM, which are known to generate acidic intracellular environments and would then shift the ionic equilibrium further in favor of more free La^3+^ ions^22^.

We hypothesized a possible mechanism for the cytotoxicity of La_2_O_3_ NPs in GBM cells was the generation of ROS by free La^3+^ ions^23^, given the positive correlation we found between ROS markers and NP concentrations across our PDCLs. This hypothesis was supported by our series of pretreatment ROSSc experiments which showed reversal in cell viability and western blotting trends indicating a degree of dependency on ROS for the reported observations. Associations between lanthanum and ROS have been previously reported in the literature, although examples of La_2_O_3_ specifically are not common. One study was able to show that La_2_O_3_ NPs in the presence of antioxidant ascorbic acid caused less cell death, indirectly implicating ROS as a main effector of cell death^24^. Otherwise, free La^3+^ ion studies have been able to correlate the presence of lanthanum with corresponding increases in ROS within astrocyte and other cancer cells^25,26^.

The significance of our finding is that ROS plays a major role in the induction of apoptosis by intrinsic and extrinsic pathways that can be reversed in the presence of ROSSc^23^. There is anecdotal evidence that free La^3+^ ions specifically activate the intrinsic, mitochondrial pathway in cancer cells^27,28^, which supports the more general notion that the intrinsic pathway appears to be the most relevant pathway in NP-induced cell death^29^. Much of the effect by ROS is thought to be mitochondria-mediated via this pathway, as implicated by our Mitosox findings. In terms of the extrinsic pathway, the generation of ROS as a trigger via lanthanum has not been proposed to date as of yet. There are no reports in the literature associating lanthanum-based NPs to the extrinsic pathway, although other REE free ions such, as gadolinium^30^ and yttrium^31^ in other tumor types, have been linked with the extrinsic apoptosis through ROS generation.

In addition to apoptosis, which occurs outside the nucleus, our results suggest that these NPs may also cause direct nuclear damage based on our electron microscopy and western blotting results. This is another novel finding of ours, as to date, there is no evidence in the literature associating lanthanum in any formulation directly with ɣ-H2AX generation in vitro. The closest would be a study by Paiva et al.^32^, who utilized a comet tail assay to demonstrate that free La^3+^ ion was associated with DNA damage in normal Jurkat cells. Furthermore, our results also suggest autophagy to be another alternative apoptosis-independent pathway to cell death in this setting^33^. With respect to GBM, there have been in vitro studies previously reporting autophagy after the use of NP therapy^34,35^, however, these were not by lanthanum or other REE NP therapies. Autophagy has however been previously reported with La_2_O_3_ NP in non-cancerous cells^36^.

Lanthanum-based NPs with radiation studies have not been reported previously in GBM, but have been shown to successfully enhance ionizing radiation in other cancers^37^. Although colony formation assay is vulnerable to observation bias due to possible colony overlap, we note that trends we report would still persist, albeit it at higher absolute proportions. Nevertheless, the trends we report are consistent with what one would expect regarding the Auger effect^12^, where REEs emit greater energy than incoming radiation energy due to an electron cascade across their large number of orbitals^38^. Additionally, the maximum mass absorption coefficient of lanthanum to water ratio was 40 keV per National Institute of Standards and Technology (NIST) data^39^, which is comparable to other radiosensitizing metal nanoparticles^40^.

This ability to increase potency of incoming radiation is pivotal to translational efforts in the future given radiation therapy remains a staple of GBM treatment, and currently, efforts to reduce administered dose are also a competing priority in clinical studies^41^. To elucidate the exact nature of the interaction between RT and lanthanum-based NPs, intensive investigation across time and dose will be required, as whether or not these NPs have a specific synergistic radiation threshold to exert an Auger effect is beyond the scope of this study^12^. It is foreseeable however that if a small NP dose can augment incoming radiation, its use in the clinic may possibly be to reduce radiation exposure to the GBM patient rather than increase toxicity to the cancer and the patient.

Above Fa 50%, we observed synergy across all GBM PCDLs between La_2_O_3_ NP and TMZ. This is clinically important as the goal of treatment for GBM is to affect as many cancer cells as possible, if not all, meaning we desire an effect at the highest fraction possible. A synergistic association between any formulation of lanthanum, or REE for the matter, and TMZ has previously not been reported. However, given how many cytotoxic therapies in GBM treatment utilize components of the apoptosis pathways, the validity of this finding is plausible^42^. Similar synergy studies in GBM with other apoptosis-dependent therapies have been performed by Hanif and colleagues, who have investigated the drug verapamil^43^ as well as a synthetic acetamide^44^ in separate combinations with TMZ. They observed similar combination index results to indicate synergy, and noted that when in combination, bcl-2 expression was decreased, which parallels our observations with La_2_O_3_ NPs. Similar to RT, reductions in patient exposure to chemotherapeutic TMZ remain a clinical priority which may be addressed using a synergistic NP therapy such as La_2_O_3_ based on our findings^45^.

A major task ahead is to evaluate the biocompatibility of La_2_O_3_ NPs in a healthy living model, as well as feasibility of NP therapy in penetrating living BBB. Our preliminary trial to detect lanthanum in mouse brain was successful as early as one hour after tail vein injection, suggesting that these NPs can indeed overcome the BBB. However, whether or not the concentrations we achieve here are sufficient to target GBM in vivo remains to be seen. We note the concentrations reported in vivo were degrees of magnitude lower than the concentrations utilized in vitro. On one hand, it may indicate that dosing in the future will need to increase in order to increase NP concentration in the brain. But on the other hand, it is comforting to know we can start initial translational studies at brain concentrations below the human brain and astrocyte toxicity thresholds and titrate upwards^9,10^. In vivo testing with orthotopic xenograft models will best replicate the brain microenvironment to ascertain how specific these NPs are to GBM lesions versus normal brain matter in a biological system, and how they may interact differently with the adjuvant therapies described. This will also validate the plausibility, safety and pharmacokinetics of these NPs needed to reach the brain at therapeutic dose.

Furthermore, the possibility of enhancing or refining the NP therapy should also be considered. One shortcoming of this therapy in its current form is that the NPs were not produced for purposes of therapy. If NPs could be produced with greater uniformity, and indeed optimize size, shape and charge, than it is hypothesized that any potential therapeutic effects will be greater^46–48^. Lim et al.^49^ observed greater in vitro cytotoxicity with smaller La_2_O_3_ NPs compared to larger ones, indicating that size is an important factor to consider. Additionally, NPs can also act as coated vectors, with coatings such as TMZ^50^ and surfactant agents^51^ being reported in GBM cells to improve cytotoxicity and delivery respectively.

## Materials and methods

### Patient-derived cell lines

Available GBM patient-derived cell lines (PDCLs) RN1, GBML1 and WK1 were obtained from our collaborative research partners at the Queensland Institute of Medical Research (QIMR; Brisbane, Australia). PDCL G53 was a cell line developed by our group from GBM tissue sample provided directly from our collaborators at the Prince of Wales Hospital (Sydney, Australia) with ethics approval by the South Eastern Sydney Illawarra Area Health Service Human Research Ethics Committee (#10/121). Normal human astrocyte cells were sourced from Lonza, Australia, and cultured in Astrocyte Growth Medium with Astrocyte Medium Bullet Kit (Lonza). Cells were handled as previously described^52^.

### Treatments

La_2_O_3_ NPs were nanopowders (< 100 nm) formulated in PBS suspensions and prepared by sonication at 40 kHz for a minimum of 5 min followed by vortexing for 1 min. Temozolomide (TMZ) was reconstituted in DMSO at 10 mM, and treated for 8 days as per our established protocol previously described^53^. Maximum tested concentration of TMZ resulted in maximum final DMSO of 1% v/v which did not induce any observable cytotoxic effect on cells when tested separately. Radiation therapy (RT) was administered at 4 Gy using the X-Rad 320 Biological Irradiator (Precision X-ray, USA). Radical oxygen species (ROS) scavenger (ROSSc) N-acetylcysteine (NAC) administered at 5 mM^54^. All compounds were purchased from Sigma Aldrich (Sydney).

### Transmission electron microscopy

After PDCLs were seeded overnight in thinly Matrigel-coated 27 mm single-wells (Thermo Fisher Scientific, USA), they were exposed to the NP therapy for one hour. The media was removed and cells were fixed in 2.5% glutaraldehyde in 0.1 M sodium cacodylate buffer solution (pH 7.4) for one hour at room temperature. Cells were then post-fixed with 1% osmium tetroxide for 1 h at room temperature, and serially dehydrated in increasing percentages of ethanol (60–100%). They were then embedded in increasing ratios of LX-112 resin/ethanol to 100% resin in a BioWave microwave (Pelco, USA), and left overnight at 60 °C to polymerize. A ultramicrotome (Leica, USA; model UC6) was used to cut vertical ultrathin (60 nm) sections. These sections were then imaged using F200 transmission electron microscope (JEOL, USA; model1011) at 80 kV.

### Mass spectrometry

To determine the extent of dissociation of La_2_O_3_ NP, 1000 µg/mL solutions were prepared in Milli-Q water at both pH = 7 and pH = 4. After 72 h, samples of supernatant were collected after centrifugation for 1 h at 1500 RPM. Samples were then processed using an Elan 6100 inductively coupled plasma (ICP) mass spectrometer (Perkin Elmer Sciex Instruments, USA) to determine the amount of La^3+^ in the sample. The elemental concentrations were calculated based on stoichiometry of the NP.

### Cell viability and combination assays

The optimum cell density of each PDCL was established using the MTS, CellTiter 96 Aqueous One Solution Cell Proliferation Assay (Promega, Australia). PDCLs were treated then with increasing concentrations of drug therapy to determine the half-maximal inhibitory concentration (IC_50_) at 72-h and 8-day durations. Combination studies required PDCLs to be exposed to combinations of La_2_O_3_ NP and TMZ over a range of concentrations at 1:1 IC_50_ ratio treatment for 8 days. CompuSyn 1.0 (Compusyn, USA) was used to determine the combination index (CI) which offers quantitative definition for additive effect (CI = 1), synergism (CI > 1) and antagonism (CI < 1) of drug combinations by the Median Effects methods described by Chou and Talalay^55^. Dependent variable was fraction affected (Fa), a proportion of how many cells ultimately were affected and died.

### Colony formation assay

Colony formation assay were performed as previously described from our group, using PDCLs that could form colonies and had comparable double times; RN1 (double time 63.8 h), GBML1 (double time 61.8 h) and G53 (double time 60.5 h)^52^. Briefly, PDCLs were seeded in 6-well plates overnight, and assessed 14 days after last treatment. Wells were washed with PBS and fixed with 0.5% crystal violet dye in 1:1 distilled water:methanol for 1 h, before being washed again. Stained colonies consisting of > 50 cells were considered 1 CFU. Quantification was performed using first an automated process in ImageJ (NIH, USA), and then manual verification at high magnification^56^. All counts were normalized to the negative control for subsequent analysis.

### Flow cytometry analysis

Flow cytometry was performed as previously described^52^. Briefly, PDCLs were seeded in 6-well plates, exposed to treatment the next day for 72 h unless otherwise specified. For endocytosis studies, before 1 h exposure to 100 µg/mL NP, cells were first pre-treated for 30 min with specific endocytosis pathway inhibitors (Sigma-Aldrich); nystatin (NY, 10 µg/mL), chlorpromazine (CH, 10 µg/mL) and cytochalasin D (CD, 1 µg/mL) to inhibit caveolae- and clathrin-mediated endocytosis, and micropinocytosis respectively^57–60^. Median side scatter area (SSC-A) measure was used as a surrogate for marker for NP uptake within a cell after solitary NPs were gated out. For apoptosis and autophagy studies, Annexin V (y-axis) versus PI (x-axis) markers were used. Proportions of cells positive versus negative for both markers were analyzed after solitary NPs were gated out, and classified as viable (AnnV^−^/PI^−^), early apoptotic (AnnV^+^/PI^−^), late apoptotic (AnnV^+^/PI^+^), and necrotic (AnnV^−^/PI^+^). At the time of measurement, cells were harvested, washed once in in PBS and then fixed in 70% v/v ethanol for 30 min at 4 °C. Fixed cells were then pelleted by centrifugation, being washed twice with PBS and resuspended in 400 μL of staining solution containing 50 μg/mL propidium iodide (Sigma-Aldrich) and 100 μg/mL DNase-free RNase (Roche). Cellular markers were probed using dyes Annexin V (1:50; Roche), H2ACDFDA (10 µM, ThermoFisher Scientific) and MitoSox (5 µM; ThermoFisher Scientific). Single cell assessment was then performed via flow cytometry using a BD FACSCanto II system (BD Biosciences) for 10,000 cells from each sample, and the data obtained analyzed using the FlowJo software (BD Biosciences) utilizing the geometric mean of fluorescence intensity (H2DCFDA, 530 nm; Mitosox, 585 nm) after gating out solitary NPs.

### Western blot analysis

Western blotting was performed as previously described^52^. Briefly, PDCLs were seeded in 6-well plates and exposed to treatment. At the time of measurement, cells were lysed by incubation in RIPA lysis buffer supplemented with PMSF and protease inhibitors, and stored in − 80 °C overnight. Protein concentrations were normalized using a BCA protein assay (Pierce Biotechnology). Proteins were denatured using Laemmli denaturing buffer (Bio-Rad Laboratories) supplemented with β-mercaptoethanol and separated on a 12% polyacrylamide SDS-PAGE gel. Proteins were transferred to a PVDF membrane and immunoblotted using antibodies against bax (1:1000; Cell Signaling), bcl-2 (Cell Signaling; 1:1000) cleaved caspase-8 (1:1000; Cell Signaling), caspase-9 (1:1000; Cell Signaling). LC-I and -III (1:1000; Abcam), ɣ-H2AX (1:1000; Cell Signaling), and cleaved PARP (1:1000; Cell Signaling). To control for protein loading, membranes were probed with alpha-tubulin (1:1000; Abcam) and eIF4E (1:1000; Abcam). Membranes were divided based on predicted weight according to ladder, and then were developed using Clarity Western ECL Substrate Biorad Chemiluminescence system (BioRAD #170-506). Blots were imaged using the LAS-4000 (Fuji Photo Film Co. Ltd., Japan) at appropriate exposure times, and analyzed using Image Studio Lite (LI-COR Biosciences, USA) and ImageJ (NIH, USA).

### Animal studies

All in vivo studies in 7-week old balb/c nude mice (Animal Resource Centre, Perth) were conducted under ethics that had been approved by the Animal Care and Ethics Committee (ACEC #18/59B, UNSW), and all experiments were performed in accordance with relevant guidelines and regulations. At endpoint, mice were humanely euthanized with carbon dioxide overdose by inhalation. Administration route of NPs was intravenous (IV) injection at a dose of 5 mg/kg (saline) which has been previously shown to be safe in animal models^61^. Mice were prepared by heating under heat lump for 5 min to ensure maximum venous dilation. We utilized 0.5 mL 29G 13 mm insulin syringes (Terumo, USA) for injection, as it provided the most first attempt successes during training. One of the two major tail veins was chosen for injection, with a distal site chosen for the first attempt. If further attempts were required, the injection site was advanced in a proximal direction. Proximal advancement was only attempted twice before that vein was abandoned. If this occurred, the mouse was returned to its cage, and one hour later, similar attempts were made in the other tail vein. At endpoint, the brain was extracted and processed in 70% nitric acid (HNO_3_; Sigma-Aldrich) for 24 h at room temperature, and then warmed to 80 °C in a water bath for 2 h. Samples were cooled, diluted with MilliQ water to a total sample volume of 10 mL, and processed by means of mass spectrometry.

### Statistical analyses

All data were presented as mean ± standard deviation (SD). Comparisons of measurements between multiple variables was conducted by two-way analysis of variance (ANOVA) with a Bonferroni’s post-hoc test or students t-test, where appropriate. Tukey’s post-hoc test was applied in the case of multiple comparisons within variables. Statistical significance was two-sided and set at *P*-value < 0.05 and all analyses were performed using Prism 7.0 (GraphPad, USA).

### Ethics approval

Animal Care and Ethics Committee (ACEC #18/59B, UNSW).

## Supplementary information

Supplementary Information
