# Supplementary material for: Cytotoxic lanthanum oxide nanoparticles sensitize glioblastoma cells to radiation therapy and temozolomide: an in vitro rationale for translational studies
Source: Sci Rep. 2020 Oct 23;10:18156. doi: 10.1038/s41598-020-75372-3 (PMC7584621; doi:10.1038/s41598-020-75372-3)

**Cytotoxic lanthanum oxide nanoparticles sensitize glioblastoma cells to radiation therapy and temozolomide: An *in vitro* rationale for translational studies.**

**Authors:**

Victor M. Lu MD PhD^1,2^*, Toni Rose Jue DVM PhD^1^, Kerrie L. McDonald PhD^1^

**Affiliations:**

1. Lowy Cancer Center, University of New South Wales, Sydney, NSW, Australia.
2. Department of Neurosurgery, Mayo Clinic, Rochester, MN, USA.

***Corresponding Author:**

Dr. Victor M. Lu MD PhD

Department of Neurologic Surgery, Mayo Clinic.

200 First St. SW, Rochester, MN, 55905, United States.

Email: [lu.victor@mayo.edu](mailto:lu.victor@mayo.edu)

**Running title:** Lanthanum oxide nanoparticle in glioblastoma.

**Disclosures:** All authors report no conflicts of interest concerning the materials or methods used in this study or the findings specified in this paper.

**Approval:** Not required.

**Word Count:** 3,867

**Figure Count:** 5

**Table Count:** 0

**Supplementary:** Yes

**Keywords:** glioblastoma; nanoparticle; lanthanum; rare earth; cytotoxic; radiosensitization.

**Supplementary**

**
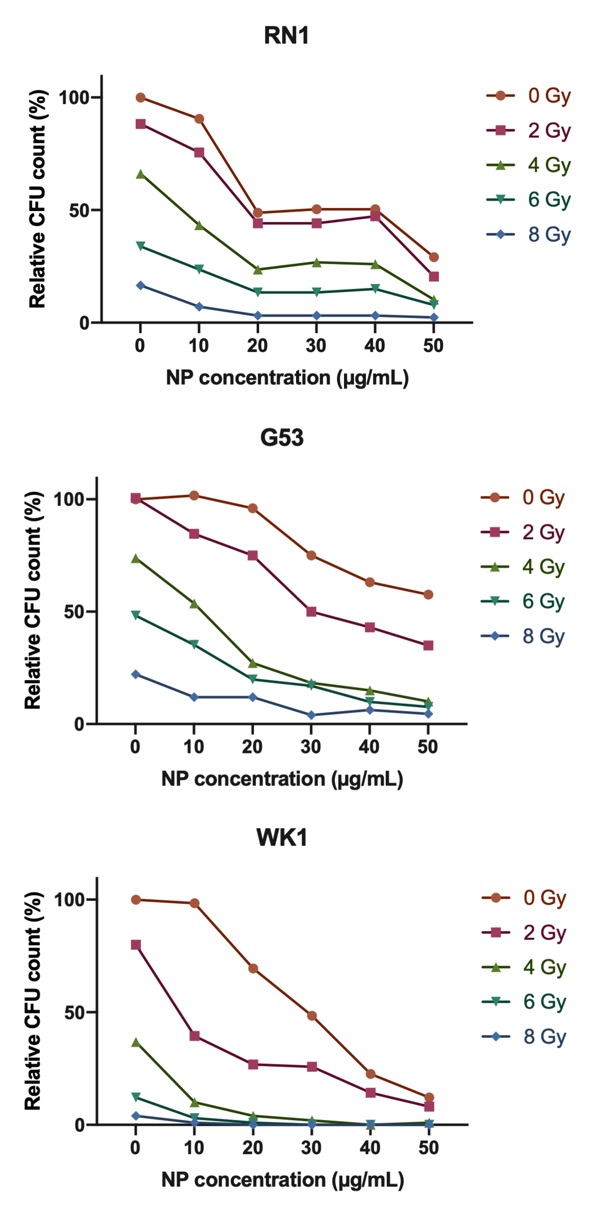
**

**Supplementary Figure 1.** Preliminary 14-day colony-forming unit (CFU) dose response curves in colony-forming patient-derived cell lines following exposure to radiation therapy (RT) 1 hour after nanoparticle (NP) administration. All CFU and fluorescence data were obtained in technical triplicate and presented as mean ± SD.


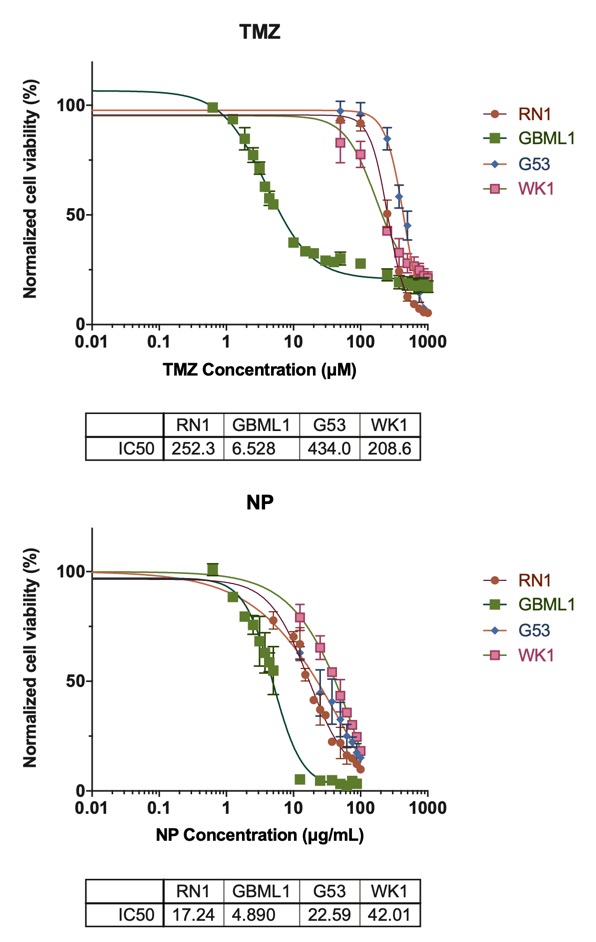


**Supplementary Figure 2.** Preliminary 8-day cell viability dose response curves in colony-forming patient-derived cell lines following exposure to temozolomide (TMZ) and nanoparticle (NP) administration. All data were obtained in technical triplicate in three independent tests and datapoints presented as mean ± SD. IC50 data presented as mean.

**Full blots**

Fig 2E, cPARP


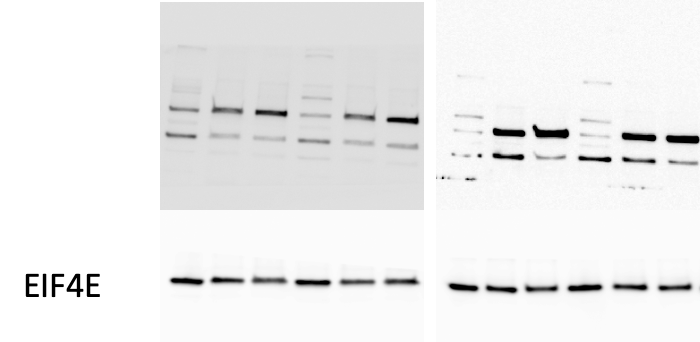


Fig 2E, Caspase-9


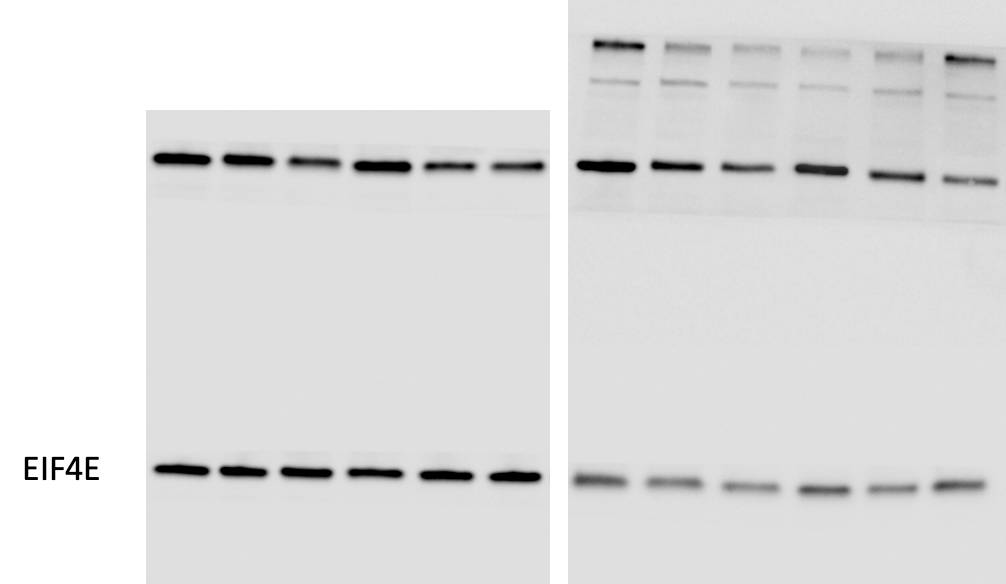


Fig 2E, cCaspase-8


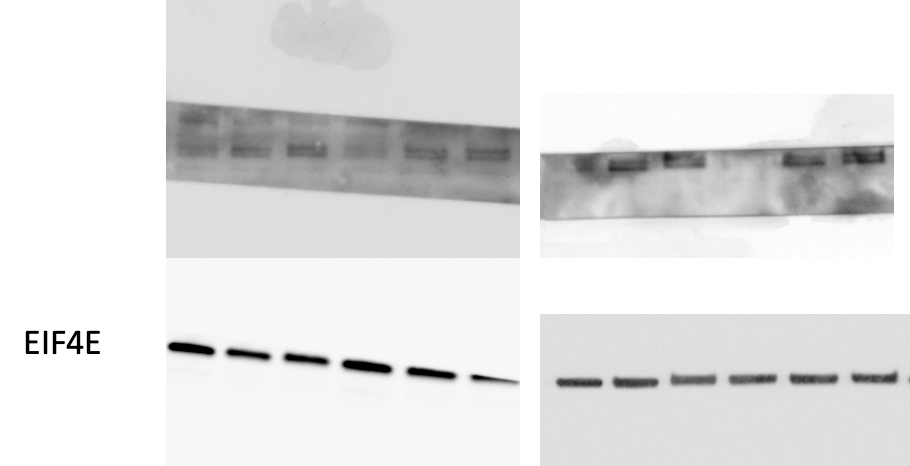


Fig 2E, bcl-2


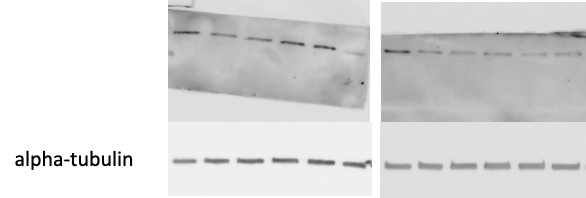


Fig 2E, bax


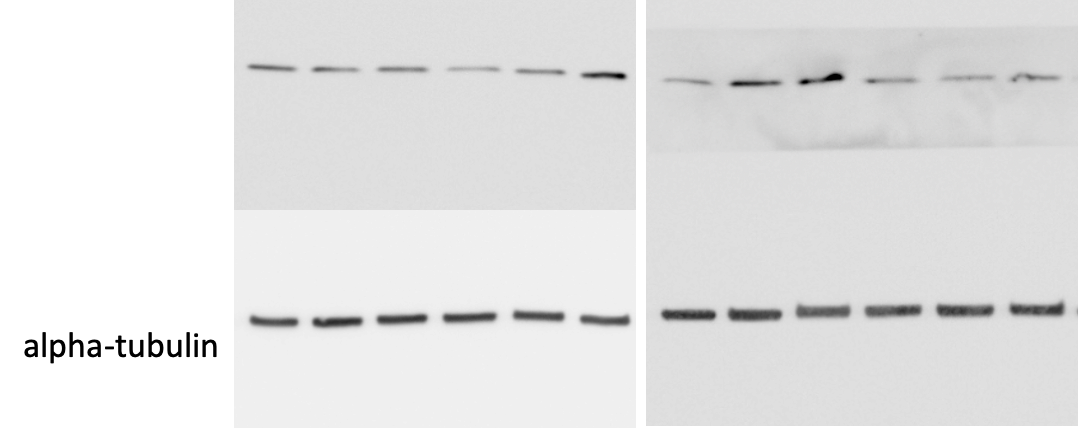


Fig 2E, H2AX


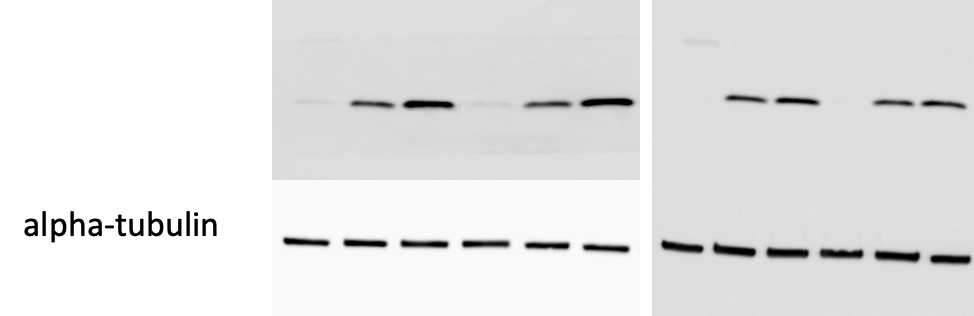


Fig 2E, LC-3


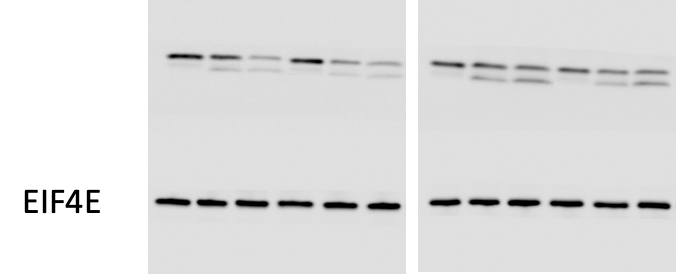


Fig 2G, cPARP


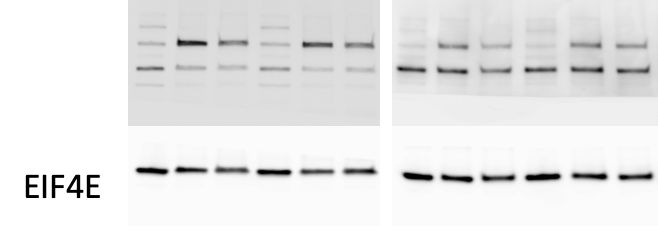


Fig 2G, Caspase-9


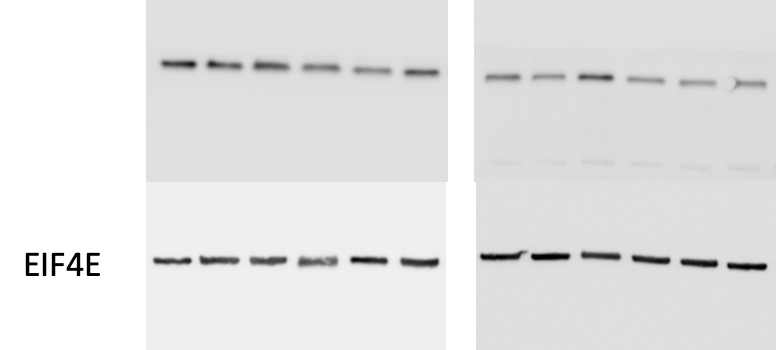


Fig 2G, cCaspase-8


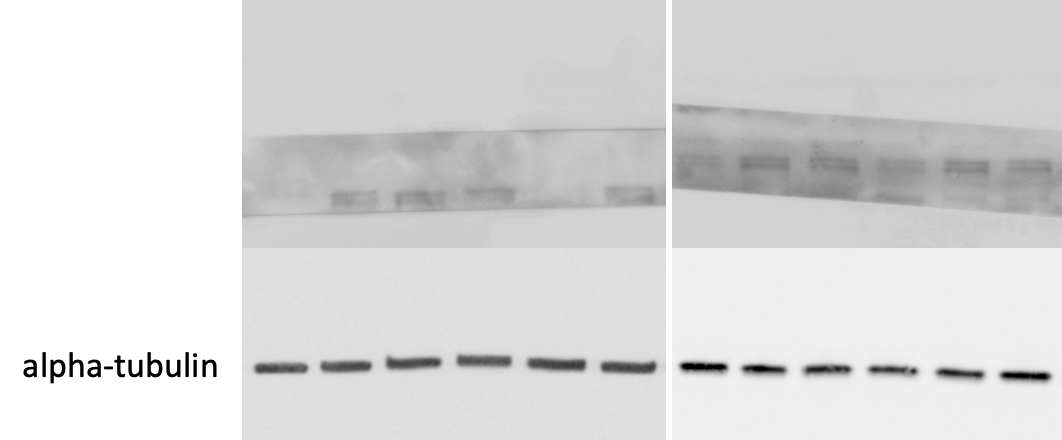


Fig 3E, H2AX


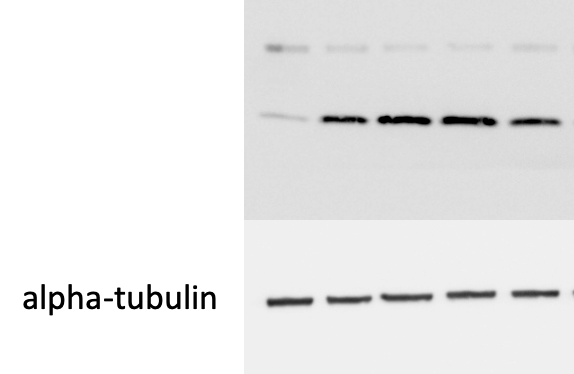


Fig 4C, Bcl-2


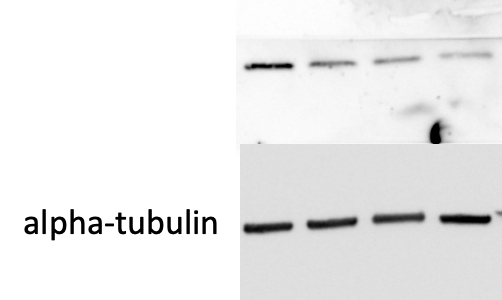

Supplement: Supplementary file 1 — Supplementary Information [file 41598_2020_75372_MOESM1_ESM.docx]
